# Coupled intra- and inter-tunnel short-range order in hollandites: a case study of mannardite Ba[(Ti^4+^)_6_(V^3+^)_2_]O_16_

**DOI:** 10.1107/S2052520626006803

**Published:** 2026-07-08

**Authors:** Ella Mara Schmidt, Marta Morana, Cristian Biagioni, Arianna Minelli, Alexei Bosak, Giovanni Orazio Lepore

**Affiliations:** a Faculty of Geosciences, MARUM and MAPEX University of Bremen Klagenfurter Straße 2-4 DE-28359Bremen Germany; b Department of Earth Sciences University of Florence Via G. La Pira 4 I-50121 Firenze Italy; c Department of Earth Sciences University of Pisa Via Santa Maria, 53 I-56126Pisa Italy; d Centro per l’Integrazione della Strumentazione Scientifica dell’Università di Pisa, Pisa, Italy; e Neutron Scattering Division Oak Ridge National Laboratory Oak Ridge TN 37831 USA; f European Synchrotron Radiation Facility (ESRF), 71 Avenue des Martyrs, F-38000Grenoble, France; University of Erlangen-Nürnberg, Germany

**Keywords:** inorganic materials, inorganic porous solids, mannardite, 3D-ΔPDF, diffuse scattering, hollandite

## Abstract

Mannardite, Ba[(Ti^4+^)_6_(V^3+^)_2_]O_16_, a mineral of the hollandite supergroup, is characterized by a tunnel structure built up from double chains of edge-sharing octahedra. It shows complex order/disorder phenomena that are often related to the occurrence of diffuse scattering, satellite reflections or both. So far, diffuse scattering features have been linked to ordering of large cations in the tunnel, but recent studies on hollandite *sensu stricto* suggested that other contributions could play an important role in determining diffuse scattering features. New single-crystal X-ray diffuse scattering data, modelling with Monte Carlo simulations and the three-dimensional difference pair-distribution function (3D-ΔPDF) approach unveil a complex scenario, involving interactions not only between the Ba^2+^ cations in the tunnel, trying to avoid each other, but also among neighbouring tunnels. Short-range order observed within and between the tunnels is coupled with framework relaxation. The possible occurrence of additional cation sites in the channel, as reported in some synthetic hollandites and electrode materials, is discussed on the basis of the diffuse scattering features and modelling. This study highlights the importance of a thorough characterization of short-range order to understand, and thus tune, the physical properties of hollandite-like materials.

## Abstract 2

Single-crystal X-ray diffuse scattering combined with Monte Carlo simulations and 3D-ΔPDF analysis reveals complex short-range order in mannardite, involving coupled Ba–Ba interactions within and between tunnels and framework relaxation.

## 1. Introduction

**Figure 1:**
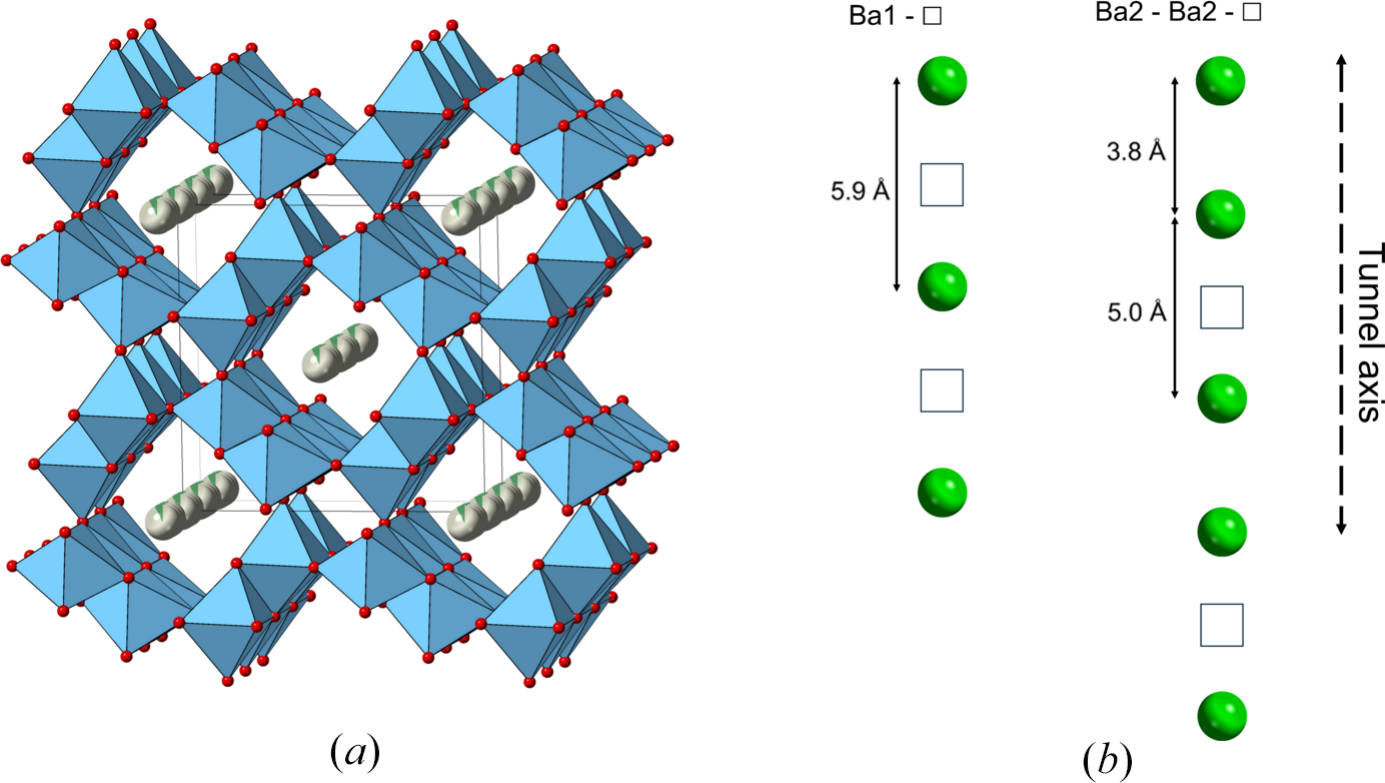
(*a*) Average structure of mannardite from single-crystal X-ray diffraction refinement and (*b*) the two proposed sequences of occupancy for the Ba sites. Ba in green, [Ti,V] in light blue and O in red.

Tunnel oxides such as hollandite-type materials have attracted considerable attention due to their ability to host ions within their channels, which allows for different applications ranging from radioactive waste immobilization (Grote *et al.*, 2019*a* ▸; Angeli *et al.*, 2008 ▸) to electrode materials (Tumurugoti *et al.*, 2021 ▸; Zeng *et al.*, 2025 ▸). They have general formula *AM*_8_O_16_, where *A* is typically a mono- or divalent cation (*e.g.* Na^+^, K^+^, Sr^2+^, Ba^2+^, Tl^+^, Pb^2+^) and *M* represents a combination of tetravalent and trivalent transition elements (Biagioni *et al.*, 2013 ▸). The tunnels, extending parallel to the crystallographic *c* axis (Pasero, 2005 ▸), are made up of double chains of edge-sharing *M*O_6_ octahedra (see Fig. 1 ▸) and can host ions of different size, charge and occupancy. Thus, partial filling and positional disorder along the tunnel direction can occur (Carter & Withers, 2005 ▸; Ishiwata *et al.*, 2006 ▸; Grote *et al.*, 2019*b* ▸), making structural disorder a common feature of such materials. In addition, the octahedral framework is relatively flexible and can locally relax in response to cation displacements, vacancies or changes in oxidation state (Morana *et al.*, 2025 ▸). As a result, many hollandites deviate from the tetragonal aristotype structure and display monoclinic distortions, occupational and displacive disorder, incommensurate modulations and complex diffuse scattering features (Post *et al.*, 1982 ▸; Carter & Withers, 2005 ▸). Such disorder is often insufficiently captured by average structure refinements based on Bragg diffraction and becomes evident through the presence of diffuse streaks, planes or satellite reflections in reciprocal space (Morana *et al.*, 2025 ▸; Carter & Withers, 2005 ▸; Grote *et al.*, 2019*b* ▸).

Minerals with such structure form the hollandite supergroup, further subdivided into the coronadite group (*M* = Mn^4+^), priderite group (*M* = Ti^4+^) and lingunite group (*M* = Si^4+^). Among them mannardite, Ba[(Ti^4+^)_6_(V^3+^)_2_]O_16_, represents an interesting case study for exploring disorder and diffuse scattering in this class of materials. In fact, the crystal structure, nomenclature and chemical composition of mannardite have been long debated. First reported from Rough Claims, British Columbia, Canada (Scott & Peatfield, 1986 ▸), it was initially described as a hydrated mineral with composition Ba[(Ti^4+^)_6_(V^3+^)_2_]O_16_·H_2_O, crystallizing in space group *I*4_1_/*a* with unit-cell parameters *a* = 14.357 Å and *c* = 5.908 Å (Szymański, 1986 ▸). This unit cell implies a doubling of the *c* axis relative to most hollandite structures. Despite the frequent occurrence of monoclinic distortions in hollandites, often associated with framework tilting or cation ordering along the tunnels (Post *et al.*, 1982 ▸; Morana *et al.*, 2025 ▸), no such distortion was reported for mannardite (Szymański, 1986 ▸). Szymański (1986 ▸) also noted that reciprocal space sections with *l* odd showed diffuse scattering as continuous sheets superimposed with weak but sharp spots, whereas no satellite reflections were observed. It was then argued that the doubled *c* axis of mannardite allows for a potentially more complex cation ordering than in a *c* ∼ 3 Å cell, where the cell edge is close to or even smaller than the ideal distance between cations, promoting partial occupancy along the tunnels, thus alternating empty and filled sites (Szymański, 1986 ▸). In the final model, two main sites and two split positions were described for Ba, allowing for a displacement from the ideal positions to minimize cation repulsion (Szymański, 1986 ▸).

A few years later, the new mineral ankangite, showing striking similarities to mannardite, was described from the Shiti baryte deposit (Ankang County, Shaanxi Province, China) (Ming *et al.*, 1989 ▸), although subsequent reassessments of the hollandite nomenclature recognized it as a variety of mannardite (Biagioni *et al.*, 2013 ▸). Interestingly, the study by Ming *et al.* (1989 ▸) described an incommensurate modulation along the *c* axis. The presence of modulation and disorder in mannardite has been further documented by X-ray diffraction (Bolotina *et al.*, 1992 ▸) and electron diffraction studies (Wu *et al.*, 1990 ▸; Xiang *et al.*, 1990 ▸). Electron diffraction patterns commonly show satellite reflections and diffuse streaks, the latter intersecting the satellite spots when present, suggesting the existence of continuous diffuse planes in reciprocal space (Wu *et al.*, 1990 ▸). These features were attributed primarily to vacancies and positional displacements of Ba atoms within the tunnels (Wu *et al.*, 1990 ▸; Xiang *et al.*, 1990 ▸). Systematic investigations across a range of hollandite compositions have shown that such modulated and disordered states are widespread, reflecting the intrinsic competition between tunnel occupancy, electrostatic repulsion of tunnel cations and structure flexibility (Carter & Withers, 2005 ▸).

Recent single-crystal diffuse scattering studies on hollandite *sensu stricto* (Morana *et al.*, 2025 ▸) demonstrate that although positional disorder of large tunnel cations such as Ba plays a dominant role in shaping the diffuse scattering, interactions between tunnel cations and the octahedral framework, as well as correlations between neighbouring tunnels, also contribute significantly to the observed diffuse scattering. Because the positional distribution and mobility of tunnel cations strongly influence functional properties, including ionic transport in Na-, Li- and Zn-ion battery electrodes (Tumurugoti *et al.*, 2021 ▸; Zeng *et al.*, 2025 ▸), and the long-term performance of ceramic hosts for radioactive waste immobilization (Grote *et al.*, 2019*a* ▸; Angeli *et al.*, 2008 ▸), a detailed characterization of short-range order in hollandite-like materials is essential.

In this work, we investigate disorder in mannardite using single-crystal X-ray diffuse scattering, combined with Monte Carlo simulations and 3D-ΔPDF analysis, which together allow us to disentangle different contributions to the diffuse scattering and directly relate real-space short-range order to framework relaxation and inter-tunnel interactions.

## 2. Experimental

### 2.1. Sample

The investigated sample was collected in the Sant’Olga level of the Monte Arsiccio mine, a currently abandoned mine formerly exploiting a small baryte, pyrite and iron oxides deposit, located in the Apuan Alps (Tuscany, Italy). There, mannardite occurs as mm-sized black prismatic crystals in veins hosted in metadolostone, along with an apatite-series mineral, anatase, arsenopyrite, baryte, dolomite, ‘hyalophane’, pyrite, quartz, sphalerite, stibnite, valentinite and zinkenite (Biagioni *et al.*, 2009 ▸).

### 2.2. Single-crystal X-ray diffraction and structure refinement

Single-crystal X-ray diffraction data for mannardite were collected using a Bruker D8 Venture diffractometer (50 kV, 1.4 mA) equipped with an air-cooled Photon III detector and microfocus Mo *K*α radiation at the ‘Centro per l’Integrazione della Strumentazione Scientifica’, University of Pisa, Italy. The detector-to-crystal distance was 38 mm. Data acquisition was performed using ω and ϕ scan modes, with 0.5° scan widths and an exposure time of 5 s per frame. In total, 1160 frames were recorded. Data integration was carried out using the *SAINT* (Bruker, 2022 ▸) software package with a narrow-frame algorithm. Corrections for Lorentz–polarization effects, absorption and background were applied using *Apex4* (Bruker AXS, 2022 ▸). Unit-cell parameters were refined based on the *XYZ* centroids of 2301 reflections with intensities greater than 2σ(*I*), within the range 5.676° < 2θ < 64.77°. The refined unit-cell parameters are *a* = 10.1506 (5) Å, *c* = 2.95560 (10) Å, *V* = 304.53 (3) Å^3^, space group *I*4/*m*. The crystal structure of mannardite was refined using *SHELXL2018* (Sheldrick, 2015 ▸). Neutral scattering curves, taken from the *International Tables for Crystallography* (Wilson, 1992 ▸), were used. After several cycles of anisotropic refinement, the conventional *R*1 factor converged to 0.0175 for 315 unique reflections with *F* > 4σ(*F*) and 24 refined parameters. The Crystallographic Information File is available as supporting information.

### 2.3. Single-crystal X-ray diffuse scattering

High-quality single-crystal diffuse scattering experiments were conducted using the dedicated diffuse scattering endstation at the ID28 beamline of the ESRF (Girard *et al.*, 2019 ▸). The crystal was glued on a kapton loop. Two complete 360° ϕ-scans were acquired at ambient conditions, with detector angles of 19° and 48°, respectively, using monochromatic X-ray radiation (λ = 0.6968 Å). Data were collected on a Pilatus3 X 1M detector operated in shutterless mode, with continuous sample rotation. Each frame was integrated over an angular range of 0.25° with an exposure time of 2 s.

Bragg reflections were indexed using *CrysAlisPro* (Agilent, 2014 ▸) to confirm phase identification, based on the average structure reported by Biagioni *et al.* (2009 ▸) and the refinement from laboratory data of a crystal from the same locality.

For diffuse scattering analysis, the raw diffraction frames were reconstructed in reciprocal space. We employed a full three-dimensional volume reconstruction, enabling subsequent 3D-ΔPDF analysis. The diffuse scattering was reconstructed using a custom Python script on a grid with − 20 ≤ *h*, *k* ≤ 20; − 5 ≤ *l* ≤ 5; Δ*h* = Δ*k* = 0.05 and Δ*l* = 0.02. The reconstructed diffuse scattering was averaged for 4/*m* Laue symmetry, yielding *R*_int_ = 0.10325. For 3D-ΔPDF analysis, layers of the reconstructed diffuse scattering corresponding to −0.2 < *l* < 0.2 for integer *l* were removed and interpolated to maintain continuity (see supporting information for a detailed description). To remove residual background scattering from air and the sample holder, an angular integration of the diffuse scattering data was carried out, employing an outlier rejection as described by Støckler *et al.* (2025 ▸). The resulting radial profile was fitted with a spline and subtracted from the reconstructed dataset.

To suppress Fourier ripples resulting from limited reciprocal space coverage, a three-dimensional Gaussian envelope was applied to the background-subtracted data, attenuating intensities at high scattering angles. The processed diffuse scattering data were then Fourier-transformed using *Meerkat* (Simonov, 2020 ▸) to obtain the 3D-ΔPDF. The structural disorder was modelled using *DISCUS* (Neder & Proffen, 2008 ▸), which was also employed to calculate the resulting diffuse scattering from the model described below.

## 3. Results and discussion

### 3.1. Average structure

The structural refinement of mannardite was carried out in space group *I*4/*m*, with *c* ∼ 2.9 Å, starting from the coordinates reported by Biagioni *et al.* (2009 ▸). The structure contains two independent Ba sites located along the tunnel axis: one site, Ba1 at *z* = 0, positioned at the tunnel centre, and the other, Ba2, slightly displaced at *z* ∼ 0.15. The resulting structure [Fig. 1 ▸(*a*)] shows an excellent agreement with previous determinations (Biagioni *et al.*, 2009 ▸) and is reported in the supporting information.

Based on crystal-chemical considerations and the ionic radius of Ba^2+^ (Shannon, 1976 ▸), two possible sequences for occupancy of the Ba sites were proposed [Fig. 1 ▸(*b*)]. The first is a ‘doublet’, consisting of an occupied Ba1 site followed by a vacancy in the neighbouring unit cell. The second is a ‘triplet’, in which a Ba2 site at *z* ∼ −0.15 is neighboured by a Ba2 site at *z* ∼ 1.15 and a vacancy at *z* = 2.0 (Biagioni *et al.*, 2009 ▸).

### 3.2. Diffuse scattering and 3D-ΔPDF interpretation

**Figure 2:**
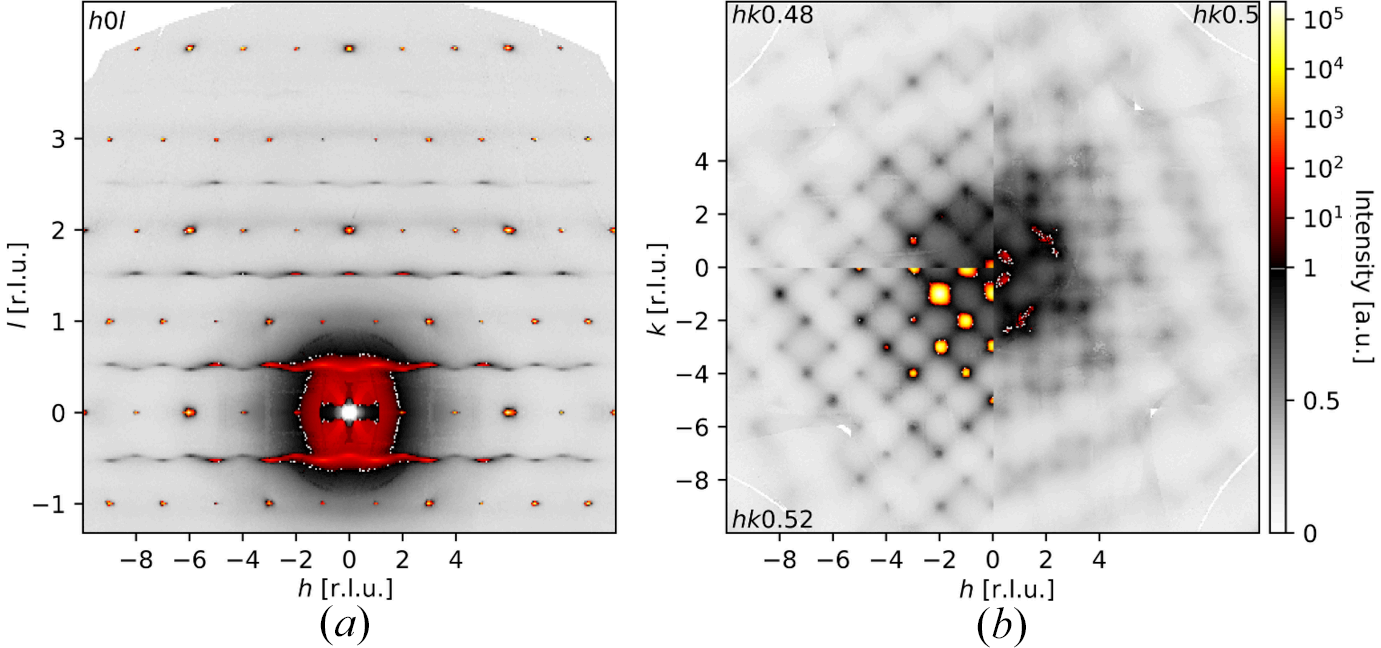
Experimentally observed diffuse scattering. (*a*) *h*0*l* layer showing wavy diffuse lines around half-integer *l*. (*b*) *hk*0.48, *hk*0.5 and *hk*0.52 layers showing the intensity distribution through the features in (*a*). Intensities are plotted on a linear scale from white to black for 0 ≤ *I* ≤ 2 and on a logarithmic scale from black to yellow for *I* > 2.

Selected reciprocal space sections, highlighting the diffuse scattering features of mannardite, are shown in Fig. 2 ▸.

The dominating feature of the diffuse scattering is concentrated in ‘wavy’ layers perpendicular to c* at approximately *l*/2 [Fig. 2 ▸(*a*)]. Additional weak, very broad, diffuse signatures are visible at *l* ∼ 2.1 and ∼ 3.1. Looking down [001], the diffuse scattering intensity is clearly not homogeneous, showing a modulation from the *hk*0.48 to the *hk*0.52 layer [Fig. 2 ▸(*b*)]. Notably a similar diffuse scattering feature, in particular satellites with diffuse scattering resembling a layer with modulated intensity, was observed in the synthetic hollandite Ba_*x*_Ti_8–*x*_Ga_2*x*_O_16_ samples with *x* = 1.33 (Bursill & Grzinic, 1980 ▸). Bursill & Grzinic (1980 ▸) related this feature to the ordering of Ba in a zigzag arrangement of vacant sites along [010], but these are separated by pairs of filled Ba^2+^, so that a sequence of a vacancy, two filled sites and a vacancy is repeated along the tunnel. This interpretation is relatively consistent with the possible filling sequences deduced from the the average structure and the diffuse scattering features of mannardite. The study by Bursill & Grzinic (1980 ▸) has the merit to be one of the first to take into account inter-tunnel correlations in hollandites, which were often overlooked in previous studies, such as the seminal work by Beyeler (1976 ▸), in favour of intra-tunnel ordering. As shown in Morana *et al.* (2025 ▸), other contributions to diffuse scattering, such as correlation between tunnels and framework relaxation, can also play a significant role in shaping the observed features. It is worth noting that waves of diffuse scattering are not confined to hollandites, but have been observed in thermoelectric materials, such as defective half-Heusler systems, characterized by vacancy ordering (Roth *et al.*, 2020 ▸; Roth *et al.*, 2021 ▸; Xia *et al.*, 2019 ▸). The 3D-ΔPDF approach (Weber & Simonov, 2012 ▸) has been proven to be an effective tool to provide insight into different contributions to short-range order (Juul *et al.*, 2025 ▸; Osborn *et al.*, 2025 ▸; Welberry & Goossens, 2014 ▸; Zhang *et al.*, 2021 ▸; Simonov *et al.*, 2022 ▸) and can thus be used to guide the analysis the local structure of mannardite.

**Figure 3:**
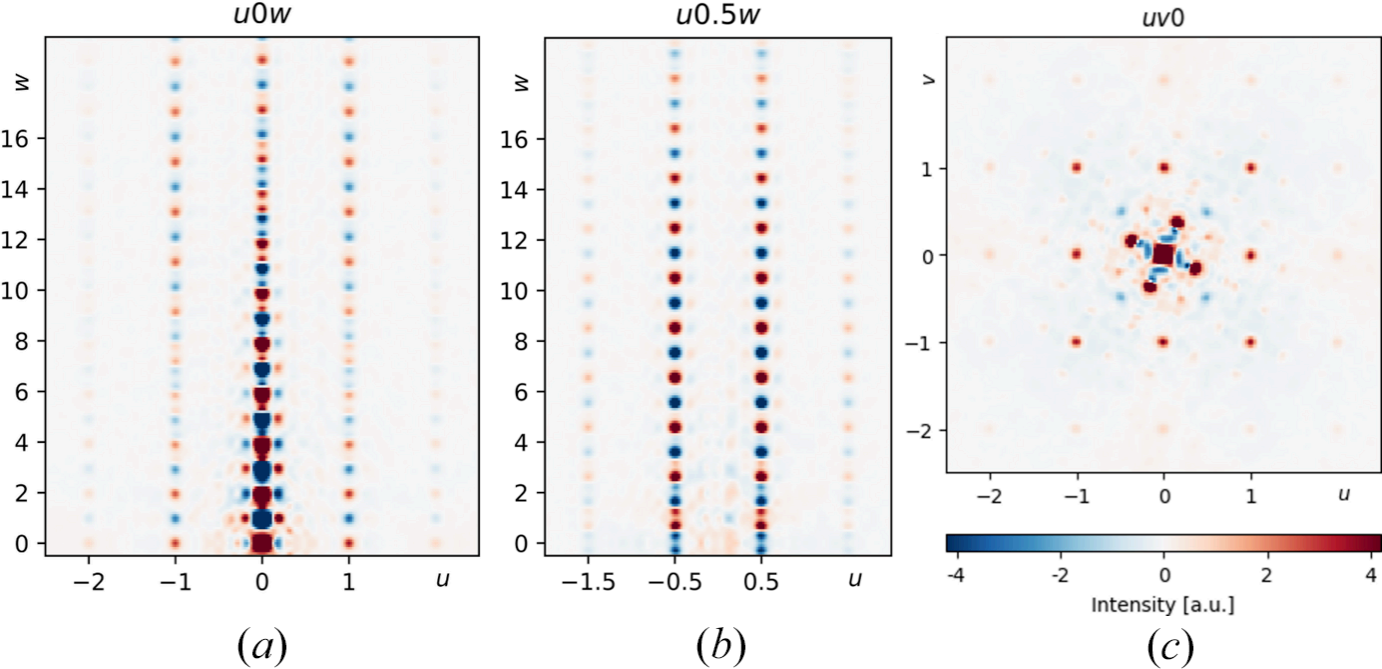
Experimentally obtained 3D-ΔPDF for the (*a*) *u*0*w* layer, (*b*) *u*0.5*w* layer and (*c*) *uv*0 layer. Negative intensities correspond to interatomic vectors in the real structure with lower electron density than in the average structure model, while positive intensities correspond to interatomic vectors with higher electron density than in the average structure model.

Fig. 3 ▸ shows selected layers of the 3D-ΔPDF obtained from the experimental diffuse scattering reconstruction. The most prominent feature is the alternation of maxima and minima along the 00*w* direction, as seen in Fig. 3 ▸(*a*). In agreement with the average structure model, this alternation strongly suggests the presence of ‘doublet’ pairs along the tunnel axis, producing a locally ordered arrangement of Ba1 and vacancies.

For larger values of *w*, the strict alternation of maxima and minima disappears, and a splitting in the positions of the maxima and minima emerges, most pronounced at *w* ∼ 13. This observation indicates that tunnels are simultaneously occupied by both ‘doublets’ and ‘triplets’, disrupting long-range coherence along **c**. Such mixed occupancy is likely for hollandite-type structures where the overall tunnel filling fraction is >1/2, while this is not observed for filling fractions <1/2 as analysed, *e.g.* by Morana *et al.* (2025 ▸).

At *w* ∼ 17, we observe an inversion of the alternation pattern: for *w* < ∼17, maxima occur at even *w* and minima at odd *w*, whereas for *w* > ∼17, this sequence is reversed. We interpret this inversion as resulting from sequences of ‘doublets’ interrupted by an odd number of ‘triplets’, producing an odd-numbered Ba1–Ba1 interatomic vector along the tunnel axis.

The 〈10*w*〉 directions exhibit a similar alternation pattern to 00*w*, although the 3D-ΔPDF intensity is significantly weaker and the splitting of maxima and minima already appears at *w* ∼ 5. This indicates that inter-tunnel correlations along 〈100〉 are mostly in phase, but the correlation length between tunnels is substantially shorter than along the tunnel direction.

In Fig. 3 ▸(*b*), the *u*0.5*w* layer reveals correlations between tunnels separated by the *I*-centring vector 〈0.5 0.5 0.5〉. In contrast to 〈100〉 in Fig. 3 ▸(*a*), a clear separation between maxima and minima is directly observed here, with no Ba–Ba pairs found at this interatomic vector. Moreover, the maximum is shifted to a larger value of *w* (*w* ∼ 0.65), consistent with an inter-channel interaction: if a Ba1 atom in a ‘doublet’ is centred at (0, 0, 0), then the neighbouring tunnel preferentially hosts a ‘triplet’ with Ba2 atoms at (0.5, 0.5, 0.65) and (0.5, 0.5, −0.65).

This interpretation agrees with the observation in Fig. 3 ▸(*c*), where a characteristic size-effect feature appears at the tunnel–framework cation interatomic vector ∼〈0.17, 0.35, 0〉. This feature suggests that Ti/V framework cations are displaced away from Ba atoms occupying tunnel centres, consistent with the behaviour reported for hollandite from the Nancy mine (Morana *et al.*, 2025 ▸). Such Ba-induced framework relaxation reduces the effective tunnel diameter in neighbouring channels, making it energetically unfavourable to simultaneously occupy Ba1 sites in both a tunnel and its *I*-centred neighbour, or a Ba1 site in one tunnel at (0, 0, 0) and a Ba2 site in the *I*-centred neighbour at 〈0.5, 0.5, 0.35〉.

In summary, the 3D-ΔPDF reveals the following key structural relationships, which form the basis of the disorder model described in the next subsections:

(i) Tunnels are occupied by Ba1 ‘doublets’ and Ba2 ‘triplets’.

(ii) In neighbouring *I*-centred tunnels, ‘doublets’ and ‘triplets’ align such that if a Ba1 in a ‘doublet’ is centred at (0, 0, 0), a ‘triplet’ is favoured in the neighbouring tunnel with Ba2 atoms at (0.5, 0.5, 0.65) and (0.5, 0.5, −0.65).

(iii) Along 〈100〉, Ba occupancies are positively correlated, indicating an enhanced likelihood of finding similar neighbours. However, this correlation is significantly weaker than the intra-tunnel correlation.

(iv) The framework exhibits a relaxation response similar to that observed in hollandite (Morana *et al.*, 2025 ▸).

### 3.3. Disorder model generation

To reproduce the structural features observed in both reciprocal space and Patterson space, we constructed ten model crystals, each measuring 10 × 10 × 100 unit cells — corresponding to 200 individual tunnels. The model generation proceeded through several stages, as detailed below.

#### 3.3.1. Intra-tunnel ordering

The results of the structural refinement indicate an average Ba occupancy of 0.58 atoms per formula unit per tunnel site. For a tunnel of 100 unit cells along **c**, this average can be realized with 16 ‘triplets’, each containing two Ba atoms, and 26 ‘doublets’, each containing one Ba atom, yielding 58 Ba per 100 unit-cell tunnel.

The 3D-ΔPDF cut along 00*w* serves as the fingerprint of intra-tunnel Ba ordering. To quantify this ordering, a reverse Monte Carlo (RMC) simulation was performed using the *DISCUS* program (Neder & Proffen, 2008 ▸). A one-dimensional chain consisting of 100 unit cells along the tunnel axis was generated and populated with 16 ‘triplets’ and 26 ‘doublets’. In a single RMC cycle, one ‘doublet’ and one ‘triplet’ are swapped along the tunnel axis.

Diffuse scattering was calculated along the 00*l* direction over −5 ≤ *l* ≤ 5 with Δ*l* = 0.01, omitting Bragg reflections. From the calculated diffuse scattering, the corresponding 3D-ΔPDF along 00*w* was derived and compared to the experimental 00*w* cut. To avoid interference from disorder signatures near (0, 0, 0) in Patterson space, only the range 0.5 ≤ *w* ≤ 20 was used for fitting. Simulations were terminated after 1000 cycles.

We performed 100 independent column simulations. On average, these models showed a 4 (2)% increase in first-neighbour ‘doublet’–‘doublet’ correlations compared to a random arrangement. No statistically significant correlations were detected for second- or higher-order neighbours, despite the long-range modulation visible in the experimental 3D-ΔPDF along 00*w*. Detailed correlation data and corresponding simulated 3D-ΔPDFs of exemplary simulations are provided in the supporting information.

#### 3.3.2. Inter-tunnel ordering

The Ba–vacancy sequences generated in Section 3.3.1 were assigned at random to the 200 tunnels in the model crystal. Inter-tunnel ordering was then optimized using a direct Monte Carlo simulation implemented via a custom Fortran program.

For each tunnel, a random number between 0 and 99 was drawn to select one of the 100 RMC-generated sequences. We implemented two types of Monte Carlo moves: (1) Each tunnel sequence can be shifted along **c** using periodic boundary conditions while maintaining internal tunnel order. (2) Two tunnels could swap their sequences. The type of move was determined by a random number with 50:50 probability for moves of type (1) and type (2).

The energy term in the simulation reflects key point ii from Section 3.2: the total energy is reduced when a vacancy at (*x*, *y*, *z*) is paired with an occupied Ba2 site at (*x* ± 0.5, *y* ± 0.5, *z* + 0.65) and an unoccupied Ba2 site at (*x* ± 0.5, *y* ± 0.5, *z* − 0.65). The simulation was run at *T* = 0 for 30 000 attempted moves to achieve an optimized configuration. On average, this reflects that every tunnel structure was altered 1500 times.

### 3.4. Framework relaxation

The structure obtained from Section 3.3.2 provides a suitable Ba–vacancy model within and between tunnels. To incorporate key point iv from Section 3.2, we included framework relaxation effects, following the approach used for hollandite from Nancy mine (Morana *et al.*, 2025 ▸). Due to low scattering contrast between Ti and V, and minimal differences in Ti/O versus V/O bond lengths, *i.e.* 1.971 versus 2.007 Å (Hawthorne & Gagné, 2024 ▸), we restricted relaxation modelling to Ti as the framework cation.

Framework relaxation was modelled using a direct Monte Carlo simulation in *DISCUS* (Neder & Proffen, 2008 ▸). Initially, Ti/V ions were randomly displaced by one of the four symmetry-equivalent vectors δ = [0.020, 0.028, 0]. Additionally, all non-vacancy atoms were displaced by a random vector consistent with isotropic displacement parameters: *B* = 1 Å^2^ for non-O atoms and *B*_O_ = 2 Å^2^ for O atoms.

The Monte Carlo simulation employed a Lennard–Jones potential to favour elongated Ti–Ba distances, while Ti–Ti, Ti–O and O–O interactions were described by harmonic spring potentials with equilibrium distances taken from the average structure. The simulation was run at a Boltzmann temperature of 1, permitting displacement swaps between atoms on equivalent sites within the supercell (Ba and vacancies excluded). A total of 5.2 × 10^7^ attempted moves were performed to ensure equilibration.

**Figure 4:**
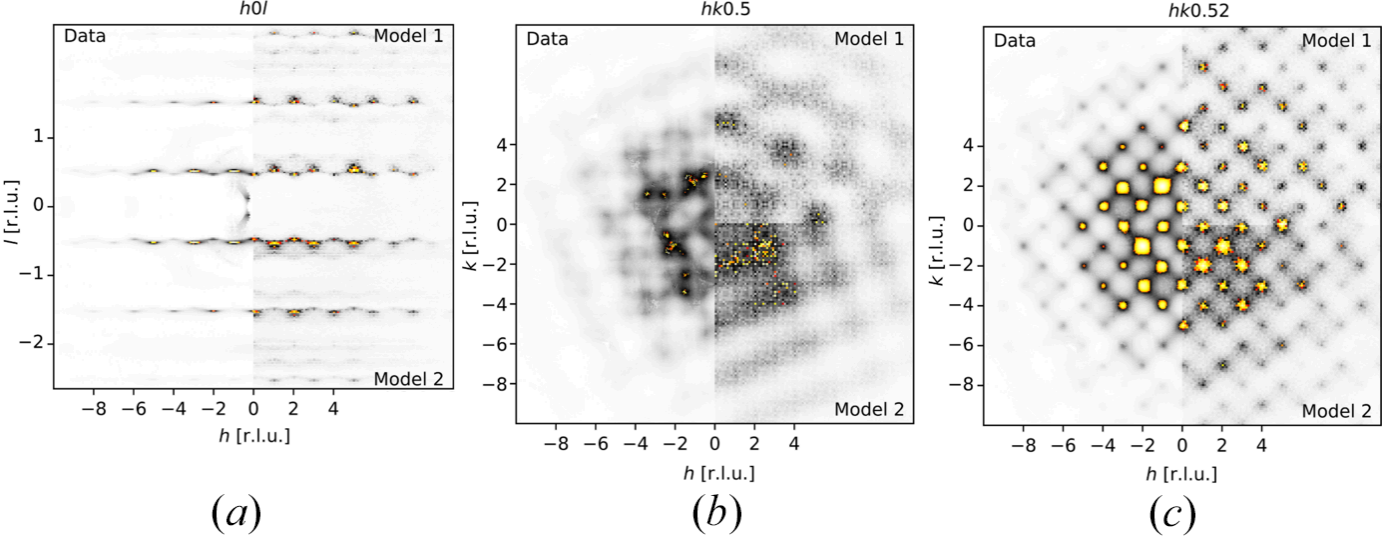
Experimentally observed and background-subtracted diffuse scattering compared with the modelled diffuse scattering.(*a*) *h*0*l* layer, (*b*) *hk*0.5 layer and (*c*) *hk*0.52 layer. Colour scale is the same as in Fig. 2 ▸.

**Figure 5:**
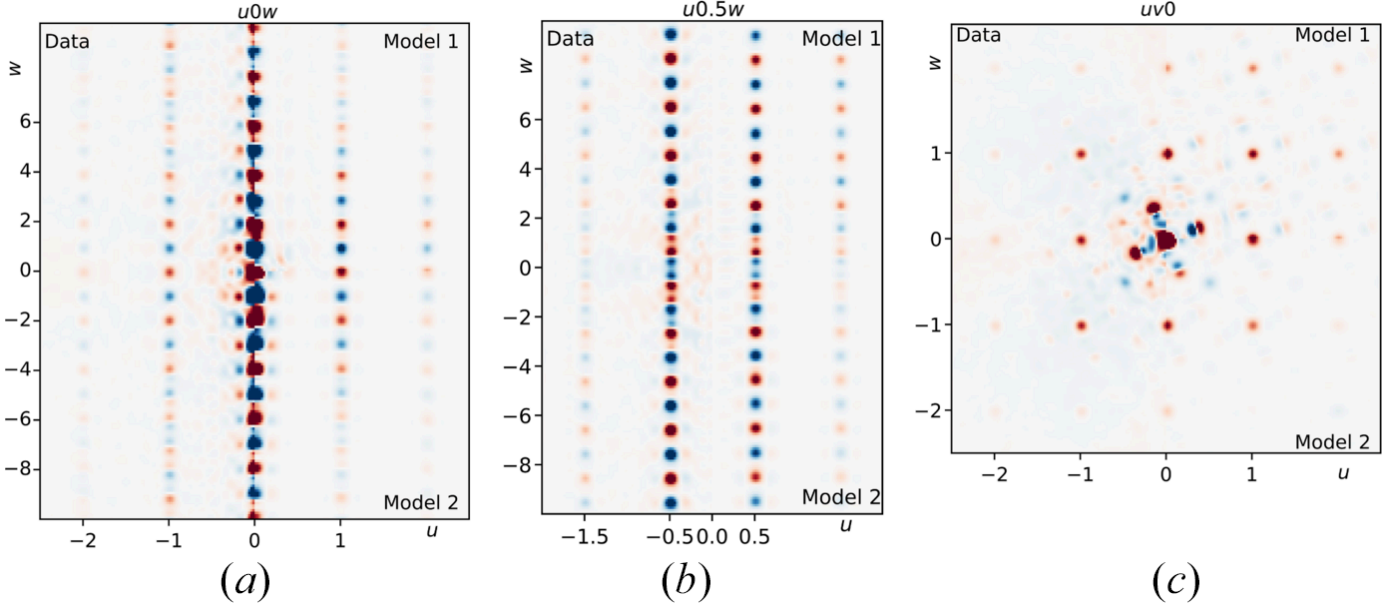
Experimentally observed 3D-ΔPDF compared with the 3D-ΔPDF of the models described in the text. (*a*) *u*0*w* layer, (*b*) *u*0.5*w* layer and (*c*) *uv*0 layer. Colour scale is the same as in Fig. 3 ▸.

Diffuse scattering was calculated using the Fourier algorithm implemented in *DISCUS* (Neder & Proffen, 2008 ▸), with a reciprocal space grid of − 20 ≤ *h*, *k* ≤ 20, Δ*h* = Δ*k* = 0.1, −5 ≤*l* ≤ 5 and Δ*l* = 0.02. The resulting diffuse scattering for Model 1 is compared with the experimental data in Fig. 4 ▸, with the *hk*0.48 layer shown in the supporting information. The corresponding 3D-ΔPDF is presented in Fig. 5 ▸.

### 3.5. Possibility of additional cation sites in the channel

Given the complexity of the features described in Section 3.2, our qualitative disorder model reproduces the principal observations well. However, close examination reveals that intensity variations in the diffuse scattering within the *hk*0.52 layer and *hk*0.5 layer are only partially captured. In Patterson space, the discrepancy is most pronounced near the 00*w* axis, where the dominant maxima and minima along the axis are accompanied by secondary extrema offset along *u* by ∼0.17.

Within the average structure model, only a Ti/V–O inter­atomic vector is located near this offset position. However, the measured intensity of this feature is too strong to originate solely from Ti/V–O correlations, particularly as other Ti/V–O vectors are not distinctly observed (for example, in the *uv*0 layer).

**Figure 6:**
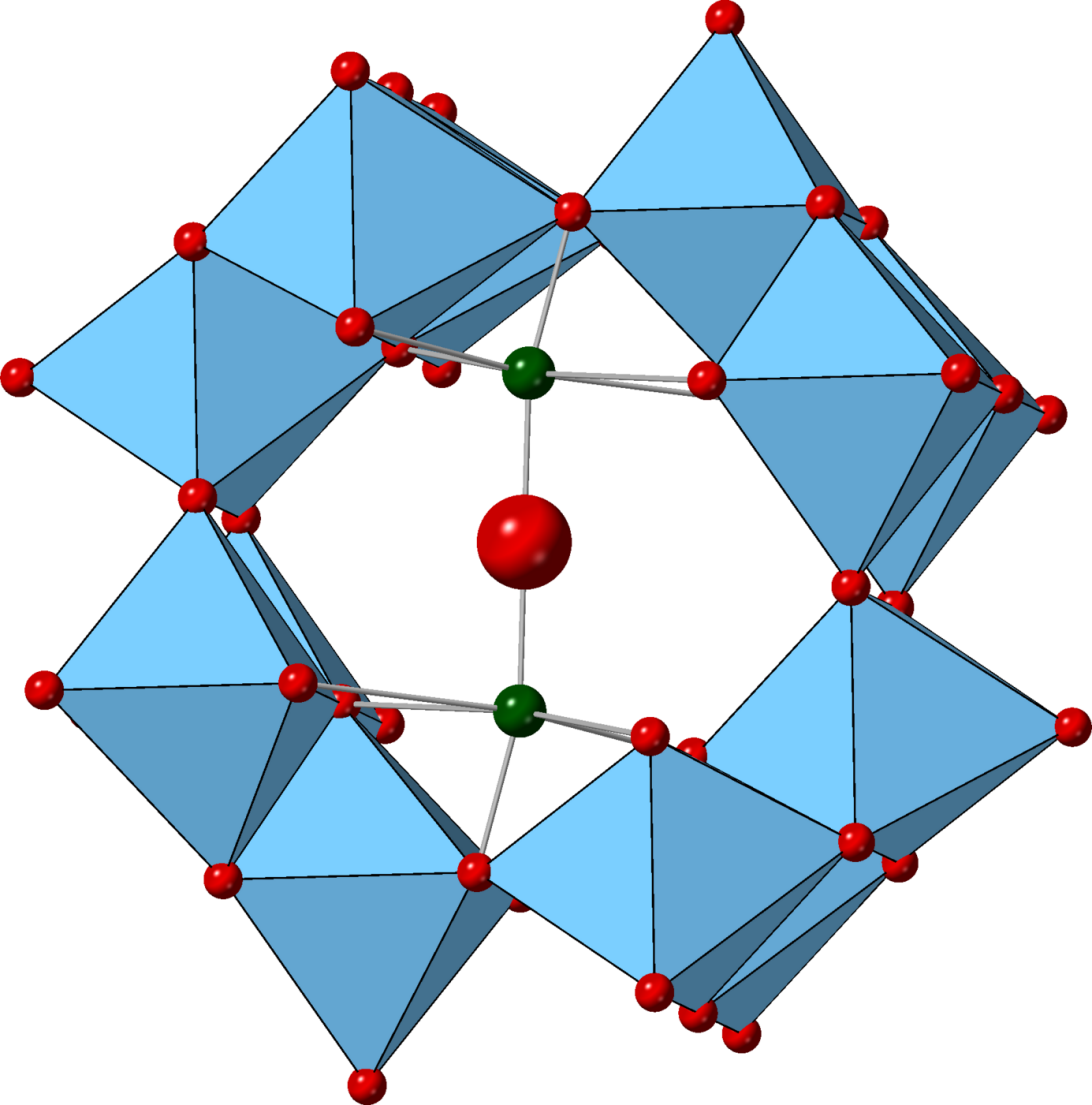
Graphical representation of Model 2, where a Ba atom in the doublet sequence is replaced by an O atom and two additional V atoms (green) are added in the tunnel.

Kanke *et al.* (1994 ▸) investigated synthetic BaV_10–*x*_O_17_ and proposed the presence of an additional V site within the tunnel. Specifically, at tunnel positions where no Ba is present, the vacancy may be occupied by an O atom, after which two additional V atoms are inserted in the tunnel, acting as ‘bridges’ between framework octahedra (Fig. 6 ▸). These extra V atoms are located at ∼(±0.17, 0, 0) in their monoclinic model, and would therefore produce the unexplained signatures observed in our diffuse scattering and 3D-ΔPDF.

To test this hypothesis, we modified our disorder model by introducing possible ‘V bridges’. In this extended model (Model 2), 20% of the vacancies within ‘doublets’ were replaced by O atoms, and two additional V atoms were inserted in the channel at either (±0.17, 0, 0) or (0, ± 0.17, 0) with equal probability. We restricted these substitutions to ‘doublets’ because, within ‘triplets’, the Ba–vacancy distances along the tunnel are shorter and likely energetically unfavourable for hosting additional V atoms.

The resulting diffuse scattering for Model 2 is shown in Fig. 4 ▸ (lower right) and the corresponding 3D-ΔPDF is presented in Fig. 5 (lower right) ▸. This extended model yields significant improvements: the intensity variations in the *hk*0.5 and *hk*0.52 layers are more accurately reproduced, and the formerly unexplained feature at (0.17, 0, *w*) in the *u*0*w* layer of the 3D-ΔPDF is now clearly accounted for. Although the difference Fourier map obtained from the structural refinement does not provide clear evidence for such a mechanism, it should be noted that the amount of V incorporated into the additional sites, when site occupancy is taken into account, corresponds to an average electron count of only ∼2 e Å^−3^. In addition, small amounts of H_2_O groups may also be present within the tunnels, leading to an overall disorder in the nature of the tunnel content, and therefore, severely limiting the detectability of ‘V bridges’ solely through the analysis of the average crystal structure via the refinement of Bragg reflection data. This additional disorder behaviour could thus be easily masked within the observed average structure.

## 4. Conclusions and implications

This study provides clear evidence that the structural complexity of hollandite-type compounds extends well beyond what is observable in the average crystal structure, as revealed by the analysis of diffuse scattering. Indeed, this approach provides direct evidence for correlated disorder involving not only cations along the tunnels, but also inter-tunnel correlations and the associated framework relaxation. The observed correlations indicate a cooperative structural response of the framework to channel occupancy; this raises the question of whether the framework response is determined primarily by the nature of the tunnel cation or rather by the degree of tunnel occupancy. In this respect, the framework adjustment mechanism in response to tunnel cations is similar to that observed in a hollandite with a total tunnel occupancy lower than 1/2 (Morana *et al.*, 2025 ▸). However, the higher occupancy observed in mannardite results in a more complex, dual distribution of cations both along individual tunnels and among neighbouring tunnels. This, in turn, imposes additional constraints on the possible modes of framework relaxation.

Our study furthermore suggests the potential occurrence of additional cationic sites, leading to a model far more complex than the description derived from the average structure only. This observation suggests that neglecting local structural effects may result in oversimplified representations of the structure. Hollandites should therefore be better described as intrinsically disordered systems, in which the real structure is defined by correlated occupational and positional disorder rather than by a purely periodic arrangement.

The observed local variations in cation distribution and framework distortion are expected to influence ion transport pathways and structural stability thereby influencing the performance of hollandite-type compounds as functional materials such as in the case of Na-, Li- and Zn-based battery applications. For instance, Tompsett & Islam (2013 ▸) demonstrate the different influence of Na and Li positioning in synthetic α-MnO_2_ materials in terms of their impact on the overall structural stability upon cycling. These considerations are even more relevant for the use of hollandites as components of ceramic matrices for radioactive waste immobilization (Grote *et al.*, 2019*a* ▸). Not only may local structural variations significantly affect ionic mobility, but specific short-range order features, such as Ti/V bridges, may additionally enhance long-term stability and leaching resistance as they could be seen as tunnel blockages. Understanding whether such features can be controlled or tuned to enhance resistance to leaching and improve durability represents an important direction for future research. A deeper crystallographic understanding of these correlated disorder mechanisms is thus essential for rationalizing the physical behaviour of hollandite-type materials and for guiding future investigations.

## Supplementary Material

Additional figures and tables, updated and extended. DOI: 10.1107/S2052520626006803/ne5020sup3.pdf

Crystal structure: contains datablock(s) I. DOI: 10.1107/S2052520626006803/ne5020sup1.cif

Structure factors: contains datablock(s) I. DOI: 10.1107/S2052520626006803/ne5020Isup2.hkl

CCDC reference: 2565334

## Data Availability

Raw experimental images, 3D-reconstructions, simulated structures and simulation scripts are available from the corresponding author upon reasonable request.
