# Supplementary material for: Coupled intra- and inter-tunnel short-range order in hollandites: a case study of mannardite Ba[(Ti4+)6(V3+)2]O16
Source: Acta Crystallogr B Struct Sci Cryst Eng Mater. 2026 Jul 8;82(Pt 4):439–46. doi: 10.1107/S2052520626006803 (PMC13470793; doi:10.1107/S2052520626006803)
Supplement: Supplementary file 1 [file b-82-00439-sup3.pdf]

STRUCTURAL SCIENCE
CRYSTAL ENGINEERING
MATERIALS

Volume 82 (2026)

Supporting information for article:

Coupled intra- and inter-tunnel short-range order in hollandites: a case study of mannardite Ba[(Ti^{4+})6(V^{3+})2]O16

Ella Mara Schmidt, Marta Morana, Cristian Biagioni, Arianna Minelli, Alexei Bosak and Giovanni Orazio Lepore

1 Average structure

Table S1: Crystal data and structure refinement

Crystal data	
Chemical formula	$Ba_{1.16}O_{16}Ti_8$
Mr	797.71
Crystal system, space group	Tetragonal, $I4/m$
Temperature (K)	293
a, c (Å)	10.1506 (5), 2.9556 (1)
V (Å³)	304.53 (3)
Z	1
Radiation type	Mo Kα
μ (mm^{-1})	8.72
Crystal size (mm)	0.07 × 0.03 × 0.03
**Data collection**	
Diffractometer	Bruker D8 Venture
Absorption correction	Multi-scan SADABS 2016/2: Krause, L., Herbst-Irmer, R.,Sheldrick G.M. & Stalke D., J. Appl. Cryst. 48 (2015) 3-10
T_{min}, T_{max}	0.318, 0.373
No. of measured, independent and observed $[I > 2\sigma(I)]$ reflections	2465, 320, 315
Rint	0.036
$(\sin\theta/\lambda)_{max}$ (Å^{-1})	0.753
**Refinement**	
$R\left[F^2>2\sigma(F^2)\right]$, $wR_2(F^2)$, S	0.018, 0.044, 1.17
No. of reflections	320
No. of parameters	24
$\Delta\rho_{max}$, $\Delta\rho_{min}$ (e Å^{-3})	0.42, -0.35

Table S2: Fractional atomic coordinates and isotropic displacement parameters (Å^2).

Fractional atomic coordinates and isotropic or equivalent isotropic displacement parameters (Å^2)					
	x	*y*	*z*	*Uiso*/Ueq*	Occ. (<1)
Ba1	0.500000	0.500000	1.000000	0.0248 (3)*	0.428 (5)
Ba2	0.500000	0.500000	0.150 (2)	0.0228 (11)*	0.075 (3)
Ti	0.16559 (3)	0.34914 (3)	0.000000	0.00884 (11)	
O1	0.29652 (12)	0.34443 (12)	0.500000	0.0088 (2)	
O2	0.16705 (13)	0.53914 (12)	0.000000	0.0102 (2)	

Table S3: Anisotropic atomic displacement parameters (Å^2).

Fractional atomic coordinates and isotropic or equivalent isotropic displacement parameters (Å^2)						
	U11	*U22*	*U33*	*U12*	*U13*	*U23*
Ti	0.01100 (15)	0.00944 (15)	0.00607 (16)	0.00152 (9)	0.000	0.000
O1	0.0096 (5)	0.0088 (5)	0.0082 (5)	0.0002 (4)	0.000	0.000
O2	0.0141 (5)	0.0083 (5)	0.0080 (5)	0.0005 (4)	0.000	0.000

2 Diffuse scattering data treatment

The crystal specimen investigated showed a slight splitting of Bragg reflections during the indexing procedure. To compensate for this, we employed a rather drastic approach of eliminating all scattering in the integer l-layers. To demonstrate that this treatment does not alter the interpretation of the diffuse scattering, we present here the data processed using a traditional punch-and-fill algorithm for comparison. The punch radius was set to 3 voxels along h, and the punched region was filled using the average of the surrounding voxels within a radius of 4 voxels along h.

The resulting $hk0$- and $h0l$-layers are shown in Figure S1. Importantly, the strongest diffuse scattering features observed in the $hk0.48$-, $hk0.5$-, and $hk0.52$-layers discussed in the main text are unaffected by this processing choice, confirming the robustness of our approach.

The 3D-ΔPDF analysis, however, reveals subtle differences when compared to our approach, as shown in Figure S2. Specifically, additional positive features appear at Ba–O interatomic vectors when the average structure is taken into account. We attribute these features to incomplete removal of the Bragg reflections by the punch-and-fill algorithm: the presence of a small monoclinic distortion modifies the average structure such that the oxygen atoms bounding the

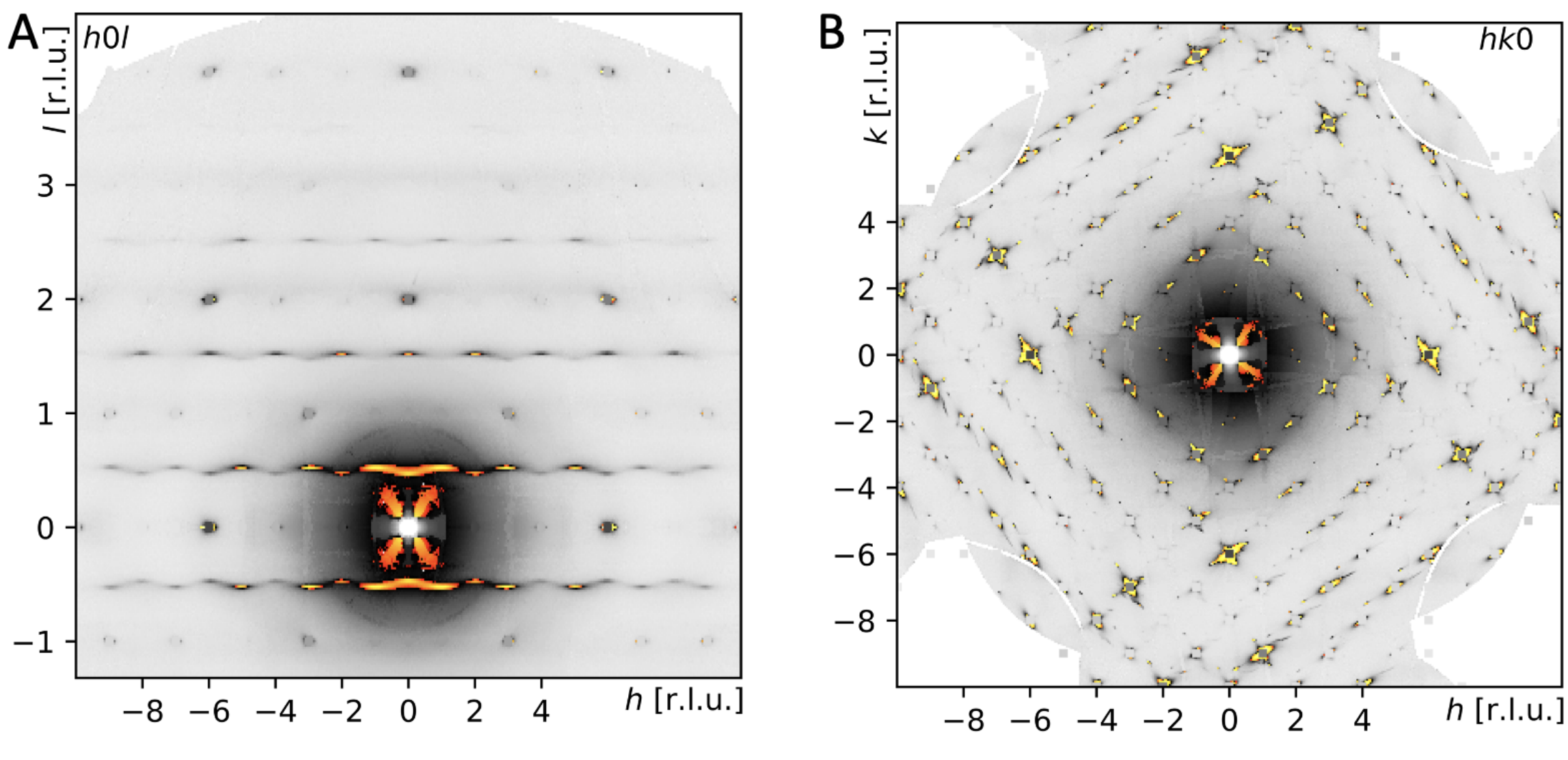

Figure S1: Diffuse scattering data treated with a classical punch-and-fill approach. (A) $h0l$-layer, (B) $hk0$-layer.

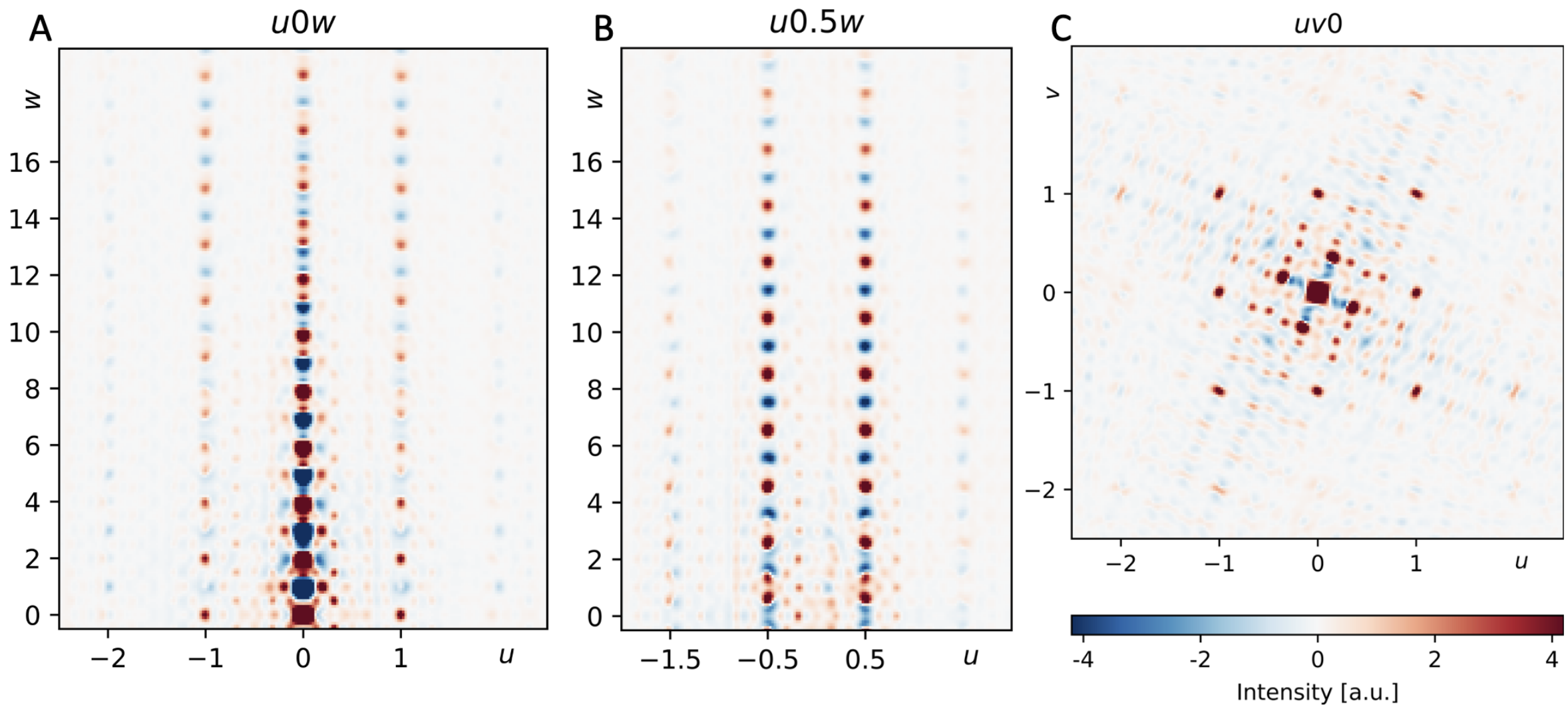

Figure S2: 3D-Δ PDF derived from the data treated with a classical punch-and-fill approach. (A) $u0w$-layer, (B) $u0,5w$-layer and (C) $uv0$-layer.

channel are displaced, giving rise to the split Bragg reflections observed in reciprocal space. This residual Bragg intensity therefore contaminates the 3D-ΔPDF, justifying our more aggressive removal of the integer l-layers.

3 RMC simulation intra-columnar order

Figure S3 shows the cut through the experimental 3D-ΔPDF along $00w$, along side the average of the ten RMC simulations that model the intra-columnar order. The correlations of the 'doublet'-'doublet' pairs as n-th neighbour within the column are listed in Table S4. The statistical probability to find a 'doublet'-'doublet' pair in an uncorrelated ordered tunnel of 'doublets' and 'triplets' is 38.3 %.

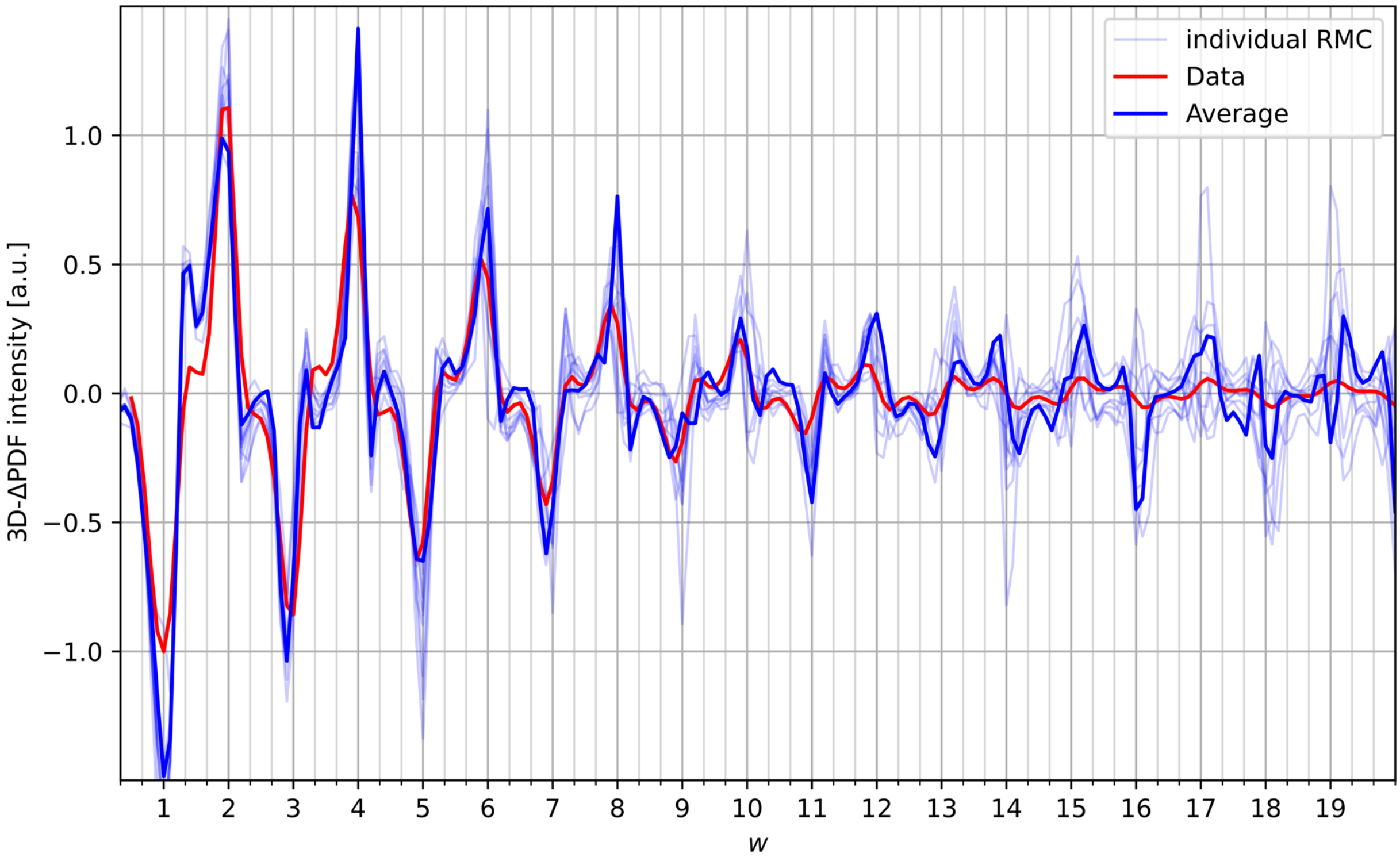

Figure S3: Experimental observed 3D-ΔPDF along $00w$ in red, individual RMC simulations in light blue, and average of the RMC simulations in blue.

Table S4: Probabilities to find 'doublet'-'doublet' pairs long the tunnel axis for the ten of the 100 individual RMC simulations.

Neighbour	1st	2nd	3rd	4th	5th	6th
Sim 0	42.9 %	42.9 %	40.5 %	40.5 %	35.7 %	35.7 %
Sim 1	45.2 %	38.1 %	38.1 %	33.3 %	35.7 %	35.7 %
Sim 2	42.9 %	33.3 %	33.3 %	33.3 %	35.7 %	38.1 %
Sim 3	45.2 %	38.1 %	35.7 %	31.0 %	38.1 %	33.3 %
Sim 4	40.5 %	35.7 %	28.6 %	31.0 %	38.1 %	42.9 %
Sim 5	38.1 %	38.1 %	40.5 %	35.7 %	38.1 %	33.3 %
Sim 6	45.2 %	45.2 %	40.5 %	40.5 %	35.7 %	40.5 %
Sim 7	47.6 %	40.5 %	38.1 %	40.5 %	40.5 %	40.5 %
Sim 8	40.5 %	35.7 %	38.1 %	38.1 %	38.1 %	33.3 %
Sim 9	45.2 %	40.5 %	38.1 %	35.7 %	31.0 %	28.6 %
Average	42.6±2.5 %	37.9±3.9 %	36.9±3.8 %	37.0±3.5 %	37.5±3.2 %	37.6±3.4 %
Deviation	4.2±2.5 %	-0.4±3.9 %	-1.4±3.8 %	-1.3±3.5 %	-0.8±3.2 %	-0.8±3.4 %

4 Framework Relaxation

The following potentials were used in the direct Monte Carlo simulation to relax the framework:

- The effective temperature was chosen as 1 in the DISCUS settings.
- 200·n_{at} (where n_{at} is the number of atoms in the simulated structure) moves were attempted.
- A Lennard-Jones potential with a depth of 100 and a minimum at 4 Å was chosen for the nearest neighbour Ti-Ba interactions.
- Spring potentials were used for Ti-Ti (target distance 3.28 Å), Ti-O (target distance 2.05 Å) and O-O (target distance 2.8 Å) nearest neighbours. A potential depth of 100 was chosen.
- The positions of the hypothesized 'V-bridges' were not relaxed.

5 Additional diffuse layers of the model simulation

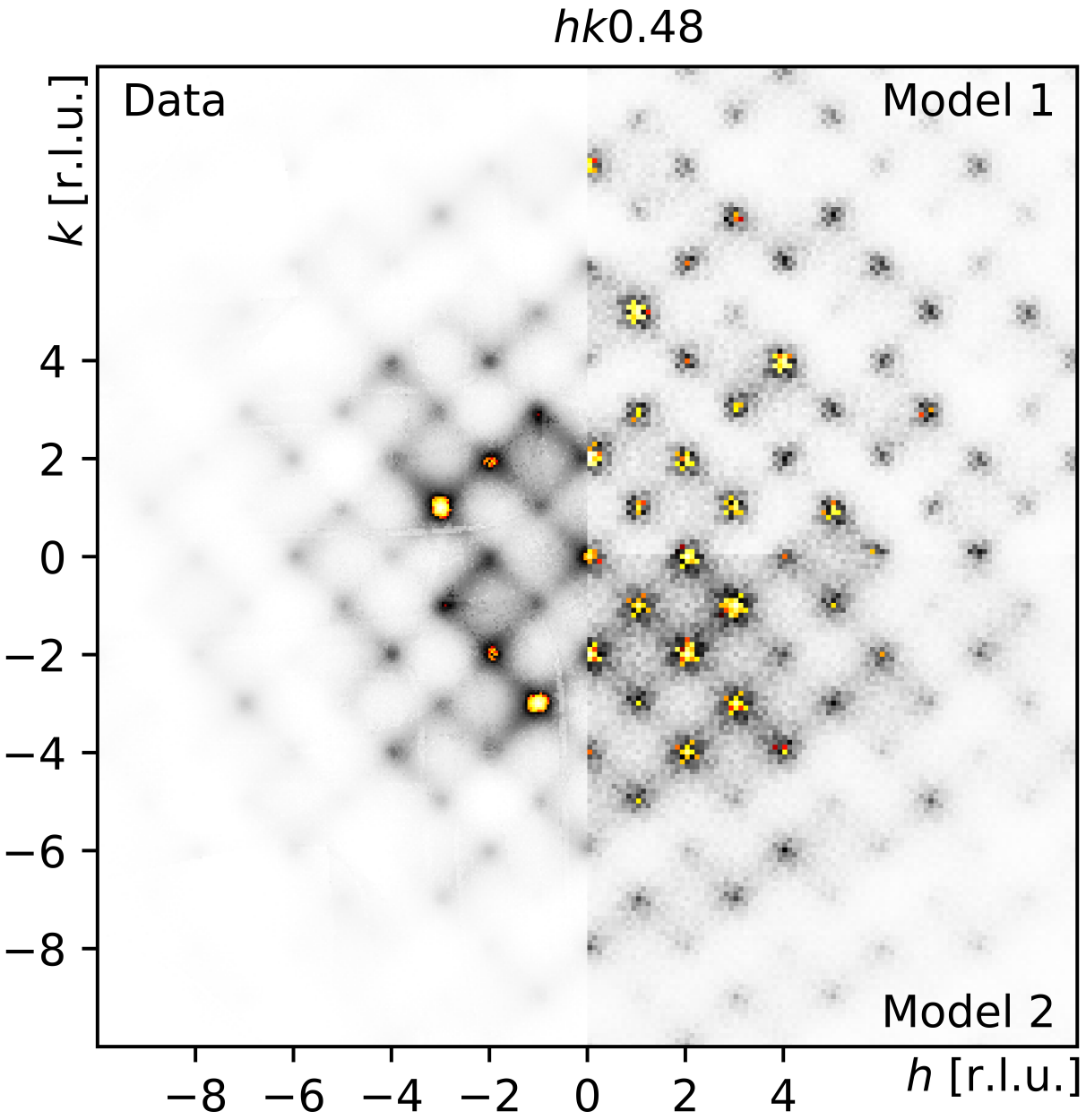

Figure S4: Experimental observed and background subtracted diffuse scattering in the $hk0.48$ layer compared to the two models described in the main text.
